# Minimal evidence for consistent changes in maize DNA methylation patterns following environmental stress

**DOI:** 10.3389/fpls.2015.00308

**Published:** 2015-05-06

**Authors:** Steven R. Eichten, Nathan M. Springer

**Affiliations:** Department of Plant Biology, Microbial and Plant Genomics Institute, University of MinnesotaSaint Paul, MN, USA

**Keywords:** DNA methylation, maize, abiotic stress, epigenetics, tissue culture

## Abstract

DNA methylation is a chromatin modification that is sometimes associated with epigenetic regulation of gene expression. As DNA methylation can be reversible at some loci, it is possible that methylation patterns may change within an organism that is subjected to environmental stress. In order to assess the effects of abiotic stress on DNA methylation patterns in maize (*Zea mays*), seeding plants were subjected to heat, cold, and UV stress treatments. Tissue was later collected from individual adult plants that had been subjected to stress or control treatments and used to perform DNA methylation profiling to determine whether there were consistent changes in DNA methylation triggered by specific stress treatments. DNA methylation profiling was performed by immunoprecipitation of methylated DNA followed by microarray hybridization to allow for quantitative estimates of DNA methylation abundance throughout the low-copy portion of the maize genome. By comparing the DNA methylation profiles of each individual plant to the average of the control plants it was possible to identify regions of the genome with variable DNA methylation. However, we did not find evidence of consistent DNA methylation changes resulting from the stress treatments used in this study. Instead, the data suggest that there is a low-rate of stochastic variation that is present in both control and stressed plants.

## Introduction

Plants, like all organisms, must respond to environmental stresses imposed throughout their life. These responses occur at all levels from fine-scale gene expression changes to large morphological changes allowing the plant to cope with environmental pressures. There is substantial interest in the potential role that epigenetics may play in plant responses to stress (reviewed in Finnegan, 2002; Boyko and Kovalchuk, 2011; Mirouze and Paszkowski, 2011; Richards, 2011). While there is evidence for a role of chromatin and small RNAs in responses to abiotic stress (Chinnusamy and Zhu, 2009; Ruiz-Ferrer and Voinnet, 2009; Gutzat and Mittelsten Scheid, 2012; Bond and Baulcombe, 2014), there is less evidence that heritable changes in DNA methylation—mitotic or meiotic–are associated with stress responses (reviewed in Gutzat and Mittelsten Scheid, 2012; Pecinka and Mittelsten Scheid, 2012; Eichten et al., 2014). DNA methylation is a highly heritable chromatin modification that can influence gene expression and transposon activity (Chan et al., 2005; Law and Jacobsen, 2010; Matzke and Mosher, 2014). There is a complex set of cellular machinery that is involved in *de novo* and maintenance DNA methylation activities (Law and Jacobsen, 2010; Matzke and Mosher, 2014) as well as a set of enzymes that can perform demethylation (Zhang and Zhu, 2012). It is possible that DNA methylation could be altered due to abiotic stress, leading to novel regulation of genes (Lukens and Zhan, 2007; Chinnusamy and Zhu, 2009). As DNA methylation is often mitotically heritable (Law and Jacobsen, 2010), it is possible that stress-induced DNA methylation changes may be maintained through mitotic cellular division allowing a continued regulation of genomic features into later stages of life.

There are a number studies that have investigated DNA methylation following biotic or abiotic stress. These studies vary substantially in terms of the treatments that have been used, the methods for assessing DNA methylation, and the interpretation of the findings. A number of studies (Lira-Medeiros et al., 2010; Tan, 2010; Verhoeven et al., 2010; Wang et al., 2011; Bilichak et al., 2012; Karan et al., 2012; Colaneri and Jones, 2013; Zheng et al., 2013) have used methyl-sensitive AFLP (MSAP) approaches to identify changes in DNA methylation in plants growing in different environments. This approach can have some limitations in quantifying DNA methylation changes (Pecinka and Mittelsten Scheid, 2012) and in many studies it was not clear whether observed changes are reproducible in multiple individuals. Genome-wide profiles of DNA methylation have provided evidence for consistent changes in DNA methylation in response to biotic stress (Dowen et al., 2012; Le et al., 2014) or tissue culture (Stroud et al., 2013; Stelpflug et al., 2014). A recent high-resolution study of both DNA sequence and DNA methylation changes in response to abiotic stress (Jiang et al., 2014) revealed elevated rates of both mutation and epimutation in response to salt stress in Arabidopsis.

Our primary goal was to search for perturbations to the maize methylome following exposure to abiotic stress. In particular we sought to test two related hypotheses. The first hypothesis suggests that there would be targeted, specific alterations of the methylome that would be reproducible in multiple individuals subjected to the same stress. The second hypothesis is that stress may destabilize the methylome and result in more variation relative to controls. In order to test these hypotheses we profiled DNA methylation in individual plants and control siblings subjected to several different stress conditions including heat, cold, and UV. While some changes in DNA methylation are observed it is not apparent that these are the result of either consistent responses to a stress or even increased instability of DNA methylation in stressed plants.

## Methods

### Plant growth conditions

Seeds from a self-fertilized B73 inbred line were planted in individual 8″ pots and grown in a growth chamber (16 h light, 26C). After 14 days, plants were randomly assigned to experimental conditions (12 plants per condition) consisting of controls grown in the greenhouse (16 h light, 26C temp), growth chamber (16 h light, 26C), heated growth chamber (50C), cold room (4C), or a growth chamber with supplemented UV light (60–64 umoles m^−2^ s^−1^) for 4 h at a time. Stress plants were maintained in growth chambers (16 h light, 26C) in between stress treatments. Stress was repeated every other day for a total of four treatments. After the stress regimen, plants were moved to a greenhouse to grow to maturity (16 h light, 26C). All plants that successfully grew to maturity and formed reproductive organs (tassel and ear formation) were used for analysis.

### Tissue harvest and meDIP profiling

Final (flag) leaf tissue was harvested and DNA was isolated using cetyltrimethylammonium bromide method (CTAB; Doyle and Doyle, 1987). DNA was prepared for meDIP profiling as described (Eichten et al., 2011). Labeled DNA was hybridized to a NimbleGen 3 × 1.4M long oligonucleotide array consisting of 1.4 million single copy probes based on the B73 reference genome sequence (RefGenv2; GEO platform GPL15621). The microarray contains ~1.4 million probes and includes a probe every ~200 bp for the low-copy portion of the maize genome (Eichten et al., 2011, 2013). Slides were hybridized for 16–20 h at 42C per manufactures guidelines. Slides were washed and scanned on the Nimblegen MS200 array scanner, aligned, and quantified using NimbleScan software (Roche Nimblegen, Madison WI) per manufacturer's instructions. This resulted in raw data reports for each of the ~1.4 M probes found on the array. Tissue culture samples were developed as described (Stelpflug et al., 2014).

### Data normalization and discovery of differentially methylated regions

Data was processed as described in Eichten et al. (2011). Briefly, raw data (pair) files were exported from NimbleScan into the Bioconductor statistical environment in R (Gentleman et al., 2004). Array data was normalized using variance stabilization normalization (vsn; Huber et al., 2002). For all samples methylated DNA immunoprecipitation (MeDIP) enrichments were estimated for each probe in a linear model accounting for array, dye, and sample effects using the limma package (Smyth, 2004). Statistical contrasts were then fit between IP samples and genomic DNA control samples (input) for each sample. Moderated *t*-statistics and the log-odds score for differential MeDIP enrichment was computed by empirical Bayes shrinkage of the standard errors with the false discovery rate controlled to 0.05. Resulting scaled and normalized methylation levels (meDIP enrichment) for each probe for each sample were used for subsequent analyses.

Two different approaches were implemented to discover differentially methylated regions (DMRs). The first approach compared the average DNA methylation level for all samples from a treatment to the average of all control samples for each probe and then utilized DNA copy (Venkatraman and Olshen, 2007) to search for segments (regions including at least three adjacent probes) with consistent differences in DNA methylation. The second approach calculated the differences in DNA methylation level for each sample relative to the average DNA methylation level for the six control plants. DNAcopy was then run to identify DMRs present in a single sample relative to the control. For both approaches, DMRs were required to exhibit at least 2-fold change in DNA methylation relative to the control average and include at least three adjacent probes. A list of non-redundant DMRs was determined by combining the coordinates for the DMRs discovered in each of the samples into a single list.

### Data access

Control, hot, cold, and UV stress microarray raw (pair) files were deposited with the National Center for Biotechnology Information GEO under accessions (GSE65266). Tissue culture data files are deposited under accession (GSE56479).

## Results

**Figure 1:**
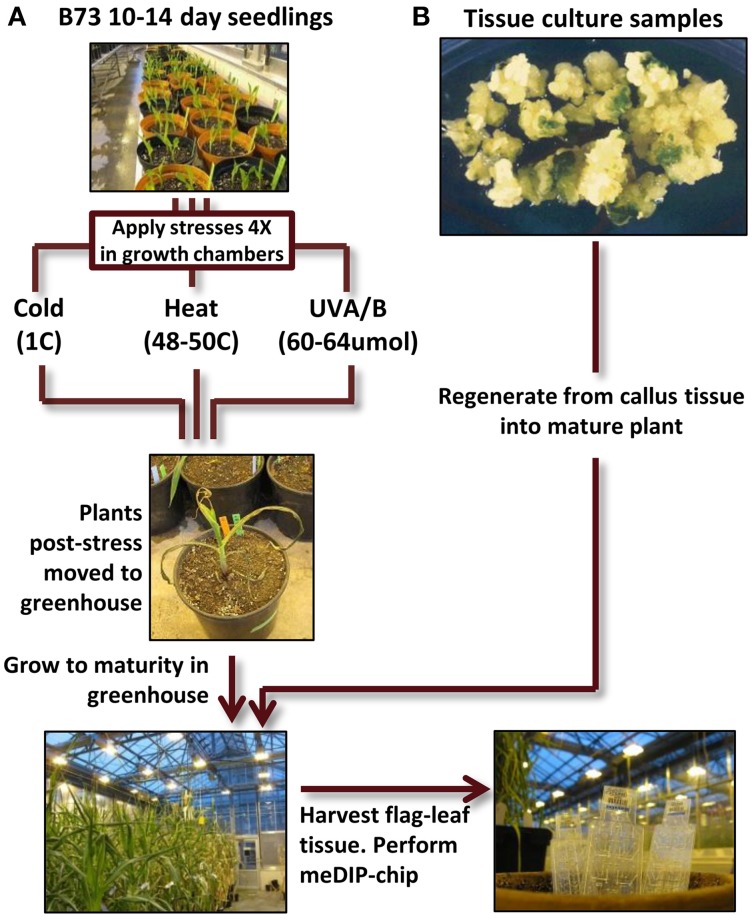
**Experimental design flow-chart. (A)** B73 seedlings (all derived from a single self-pollination) were grown for 14 days. Some of these plants were then subjected to cold, heat, or UV-B stress for 4 h per day (every other day for four treatments). At the conclusion of the treatment regime the stressed plants were then grown under standard conditions to maturity. DNA was isolated from the flag leaf (the last adult leaf initiated before the tassel) and used to perform meDIP-chip. **(B)** Tissue culture samples from the genotype A188 was used to perform regeneration. The plantlets were then grown to maturity (along with sibling non-tissue culture A188 plants) and tissue was collected from the flag leaf for meDIP-chip.

The primary set of biological materials utilized for this study was obtained from inbred B73 sibling plants (Figure 1). The inbred B73 was selected due to the availability of a reference genome sequence (Schnable et al., 2009). Sibling B73 seedlings were grown and subjected to control conditions or distinct environmental stresses 14 days after planting. The treatments included 4-h exposure to heat (50C), cold (4C) or elevated UV-A/B (60–64 umoles m^−2^ s^−1^) and were repeated every other day for a total of four treatments. The temperatures were chosen to result in severe, near-lethal, stress to maximize the potential for a response. After the final seedling stress treatment, the plants were grown to maturity using standard greenhouse conditions and DNA was extracted from the last adult leaf of the plant to use for DNA methylation profiling. In B73 the transition from vegetative to floral meristem does not occur until ~28 days after planting (Thompson et al., 2014) and therefore our profiling of DNA isolated from the last adult leaf of the plant is expected to represent the descendants of cells that had been in the meristem at the time of the stress providing the ability to search for mitotically heritable changes in DNA methylation. DNA methylation profiles were obtained for six individuals grown in control conditions, five individuals subjected to cold stress, four individuals subjected to UV stress and three heat-stressed individuals.

The DNA methylation profiles were obtained by performing meDIP followed by hybridization to an oligonucleotide microarray. This method compares the signal for meDIP samples (enriched for methylated DNA) to input DNA to gage the relative level of DNA methylation for any region of interest. This method is quite useful for detecting large differences in DNA methylation for a particular region but is limited in its ability to detect minor differences in DNA methylation (Eichten et al., 2013). In particular, this approach often finds differences in CG or CHG methylation in which one “allele” is highly methylated in these contexts and the other “allele” has low or no methylation in any context.

The DNA methylation profiles obtained for each sample were normalized and compared to each other. In general, the DNA methylation profiles obtained from these samples are quite similar to each other. The majority of the genome exhibits very similar levels of DNA methylation in all control and stress samples. Several approaches were utilized to search for DMRs. The first approach compared all samples from each treatment with all control samples to search for regions that exhibited consistent changes in methylation from a particular treatment. Very few DMRs were found when comparing all samples from a treatment to the control plants (Table 1). The number of DMRs identified using this approach is quite small with none for cold, four for heat and one for UV. This suggests that there are very few, if any, genomic regions that exhibit consistent changes in DNA methylation in response to the stresses used in this experiment.

**Table 1:** **Treatment-specific DMR identification**.

**Treatment**	**DMRs**	**#Hypo-methylated**	**#Hyper-methylated**
Cold	0	0	0
Heat	4	3	1
UV	1	0	1

Although we did not observe evidence for consistent changes in DNA methylation in response to these abiotic stress treatments it is possible that the stress treatments would result in greater rates of DMRs in individual plants. In order to assess whether the stress treatments resulted in a higher “epimutation” rate we attempted to identify DMRs that were present in individual plants. There is an inherent difficulty in identifying plant specific DMRs as we do not have biological replication since each plant it being compared as a single individual. However, our search for DMRs required that multiple probes from the same genomic region exhibit consistent changes in DNA methylation. The individual DNA methylation profiles for each plant were contrasted with the average methylation profile of the six control plants and segments (regions including at least three adjacent probes) that exhibit differences in DNA methylation were identified using DNAcopy (Venkatraman and Olshen, 2007). This allowed us to identify DMRs in a single control plant relative to the average of all controls (stochastic variation) as well as DMRs in individual stressed plants relative to the control average (stress-induced variation). A non-redundant list of DMRs that were identified in at least one individual was generated and further filtered to require a minimum of 2-fold change (Supplemental Table 1). A wide range of significant DMRs for individual plants was observed (Table 2). While there may be some biological variation in the number of DMRs there are also technical aspects that can influence the discovery of DMRs. Hybridization quality can affect signal strength and signal:noise ratios resulting in substantial differences in the numbers of DMRs that are detected.

**Table 2:** **Individual-specific DMR identification**.

**Sample**	**DMRs**	**#Hypo-methylated**	**#Hyper-methylated**
Cold_1	70	10	60
Cold_2	104	66	38
Cold_3	607	3	604
Cold_4	84	83	1
Cold_5	66	1	65
Hot_1	38	32	6
Hot_2	122	69	53
Hot_3	58	5	53
UV_1	33	3	30
UV_2	800	270	530
UV_3	9	0	9
UV_4	199	90	109
Control_1	3	3	0
Control_2	412	295	117
Control_3	284	236	48
Control_4	282	19	263
Control_5	253	2	251
Control_6	58	1	57
Non-redundant	2589	944	1935

**Figure 2:**
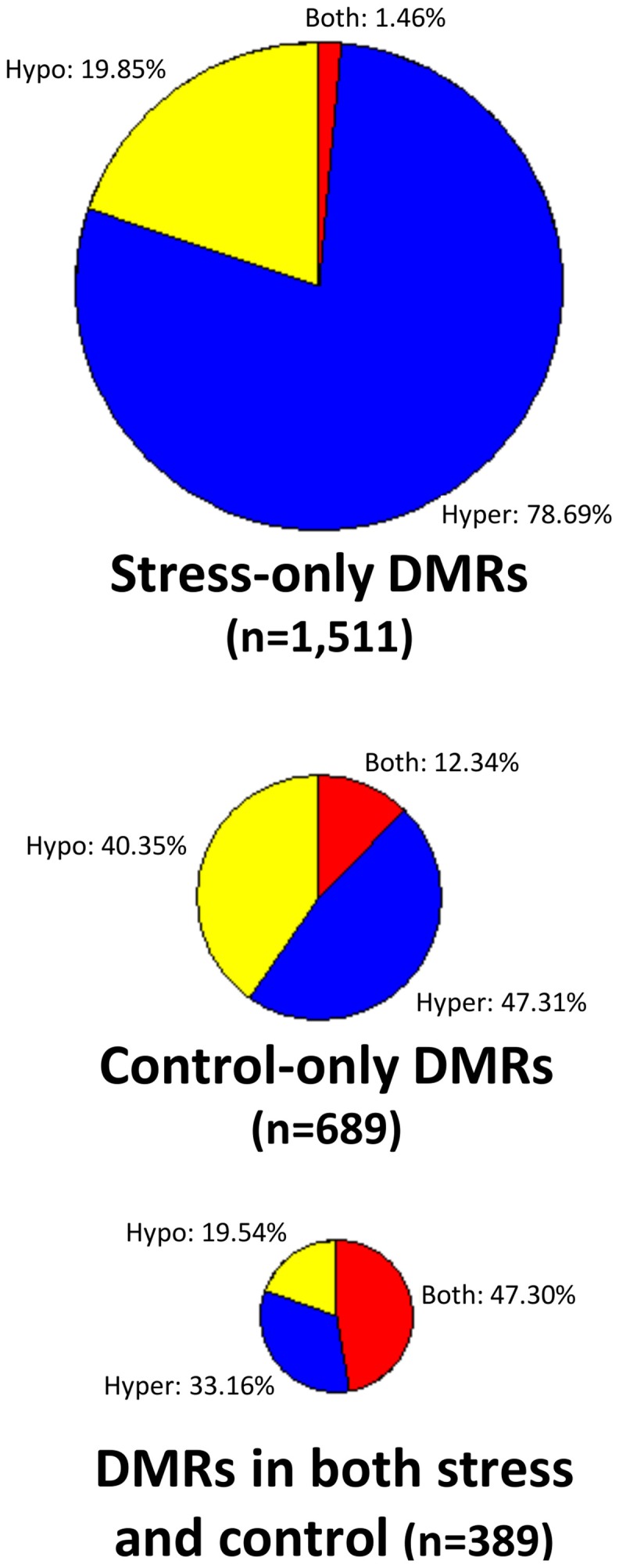
**Analysis of DNA methylation gains and losses in DMRs**. Three pie-charts (proportionally sized based on the number of DMRs for each chart) are used to show the frequency of hypermethylation (blue), hypomethylation (yellow), or both (red) relative to the control average value. The top pie-chart shows DMRs that are only present in stressed samples, the middle chart shows DMRs that are only found in control individuals and the bottom pie-chart shows DMRs that are found in both stress and control plants.

There are no clear trends in the number of DMRs or the proportion of methylation gains (hypermethylation) and losses (hypomethylation) across samples (Table 2). Overall, hypermethylation (gains compared to control average) was more prominent than hypomethylation in the full set of non-redundant DMRs. However, individual plants sometimes varied in terms of the relative number of hyper- and hypo-methylation events (Table 2). Many of the DMRs were only identified in a single individual (80% of hypomethylated DMRs and 84% of hypermethylated DMRs). However, some of the DMRs are identified in 2–5 plants (Supplemental Table 1). The DMRs were classified based on whether they exhibit a change only in the stressed plants, the control plants, or whether they change in both stressed and control individuals (Figure 2). If we limit our analysis to DMRs that are found in more than one individual plant (*n* = 618) we find that 63% are found in both control and stress individuals while 23% are observed only in stressed plants and 14% are only observed in control plants. The fact that many of the DMRs that are observed in multiple individuals are observed in both stress and control plants suggests that many of these likely reflect stochastic variation. While there are slightly more examples of DMRs in plants subjected to stress than in control plants this could be an artifact of the experimental design. This study includes 12 stressed individuals and 6 control plants so there is a greater chance for discovery in the stress group than in the control group. Given the sampling of twice as many stress plants, these numbers suggest similar frequencies of rare methylation changes in stressed and un-stressed plants. The analysis of the frequencies of DNA methylation loss and gain in these DMRs suggests some differences between the DMRs found in stress and control plants (Figure 2). The majority of the DMRs only observed in the stressed plants are hypermethylation events. DMRs that exhibit both gains and losses are quite rare in the stressed plants. In the control plants, hypomethylation events are most common.

**Figure 3:**
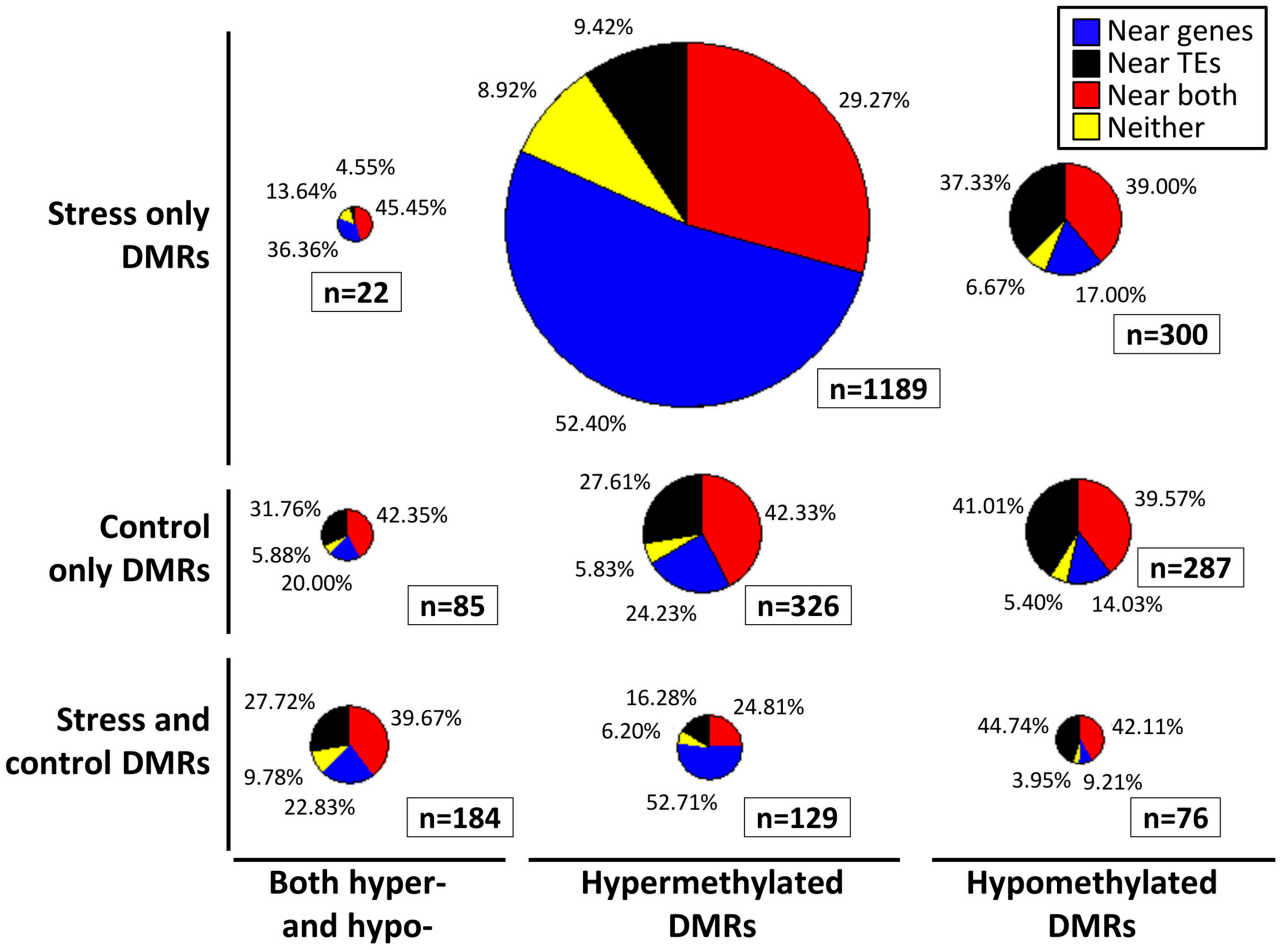
**Location of DMRs relative to genes and TEs**. Each DMR was classified based on whether it exhibits hypomethylation, hypermethylation, or both hypo- and hyper-methylation relative to the control average (x-axis). The DMRs were also classified based on whether they exhibit significant differences in stress samples only, control samples only, or both control and stress (y-axis). Proportionally sized pie-charts are used to illustrate the frequencies of DMRs located near (<500 bp) genes (blue), TEs (black), both genes and TEs (red), or neither (yellow).

The location of the DMRs relative to genes and transposable elements (TEs) was assessed (Figure 3). The DMRs were first classified based on the directionality of the methylation change (hypomethylated, hypermethylated, or both relative to the control average) and then based on whether they exhibited changes in stressed plants, control plants or both. In each of these nine groups we calculated the proportion of the DMRs located near (within 500 bp) of genes, TEs, both genes and TEs, or neither (Figure 3). The comparison of the DMRs only observed in stress plants that show either hypermethylation or hypomethylation reveals interesting differences. The hypermethylation DMRs are much more likely to occur near genes and are depleted near TEs. This may suggest differences in DNA methylation gain and loss for TEs relative to genes in stressed samples. Similar trends are observed if this analysis is restricted only to the DMRs that are observed in more than one plant.

**Figure 4:**
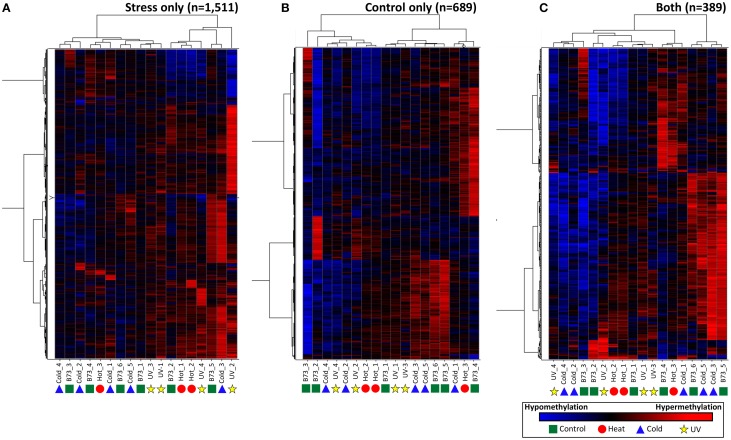
**Clustering of DNA methylation differences in plants subjected to abiotic stress**. Hierarchical clustering (Wards method) was performed for the DMRs identified in stressed plants **(A)**, control plants **(B)**, or both stress and control plants **(C)**. The heatmap uses red to indicate DNA methylation levels higher than the control average and blue to indicate lower DNA methylation levels. The samples are indicated at the bottom of each plot and the symbols indicate the treatment (green-control, blue-cold, red-heat, yellow-UV).

Although the DMRs tended to only be observed in a small number of plants it is possible that many of these DMRs show smaller changes in DNA methylation that did not reach the cut-offs used for our DMR calling. In order to assess whether there are quantitative trends in DNA methylation at these regions that are consistent in multiple plants from the same treatment we performed three different hierarchical clustering analyses using the stress-only DMRs, the control-only DMRs and the DMRs found in both stress and control individuals (Figure 4). In each case the relationships between samples are not heavily influenced by the treatments. This suggests that the small number of regions that do show changes in DNA methylation among plants do not show consistent behavior in multiple plants subjected to the same treatment.

**Figure 5:**
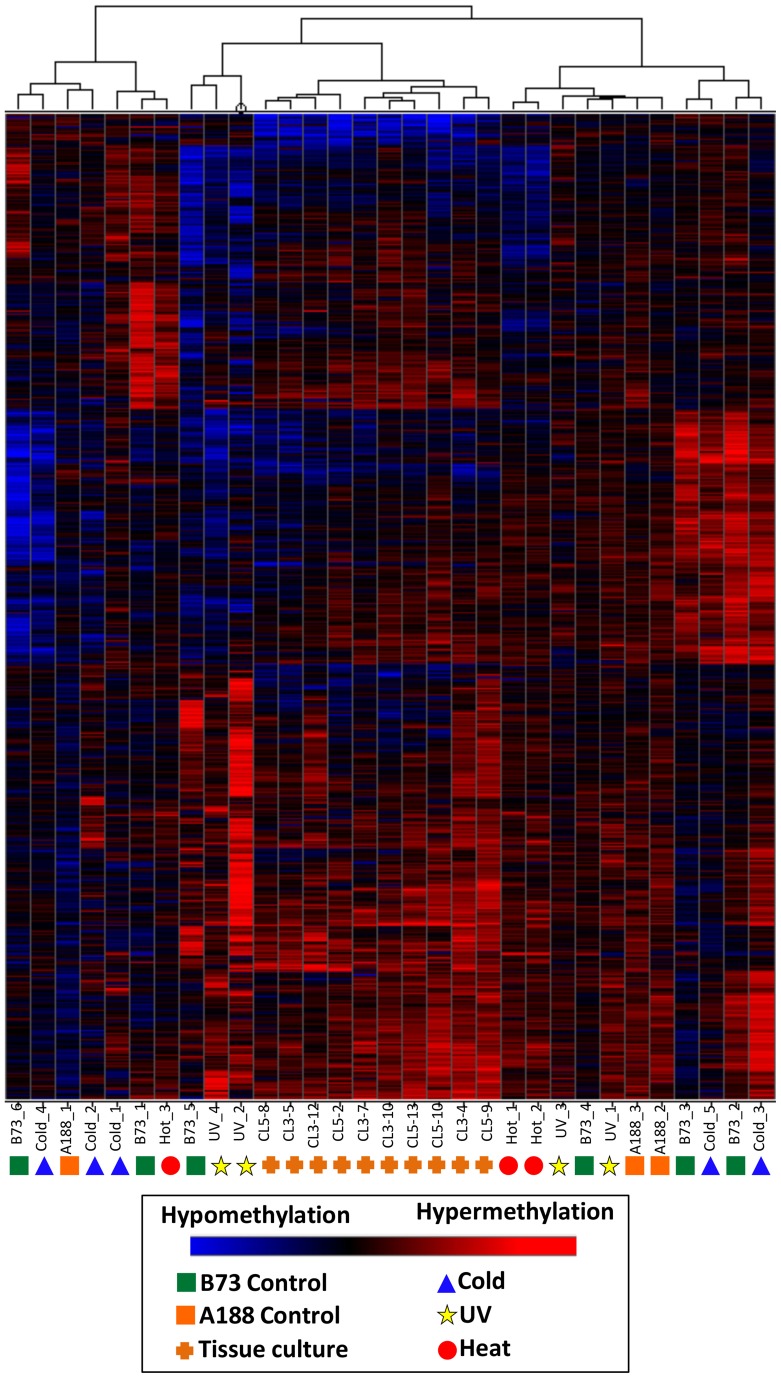
**Clustering of DNA methylation differences in plants subjected to abiotic stress or tissue culture**. Hierarchical clustering (Wards method) was performed for the DMRs identified the analysis of individual plants subjected to abiotic stress or tissue culture. These DMRs are the subset that do not exhibit differences in B73 (used for abiotic stresses) relative to A188 (used for tissue culture). The heatmap uses red to indicate DNA methylation levels higher than the control average and blue to indicate lower DNA methylation levels. The samples are indicated at the bottom of each plot and the symbols indicate the treatment (green squares, B73 control; orange squares, A188 control; blue triangles, cold; red circles, heat; yellow stars, UV; orange pluses, tissue culture regenerants).

A separate clustering was performed that included DNA methylation profiles from individual plants subjected to tissue culture and their appropriate controls (Figure 5). In previous work (Stelpflug et al., 2014) tissue culture was performed using the A188 inbred resulting in the generation of multiple R_0_ plants. The same tissue (last adult leaf) was harvested from 10 plants subjected to tissue culture and three control sibling plants that were not subjected to tissue culture (Figure 1). DNA methylation profiling and DMR calling was performed using the same methods used for the abiotic stress analysis. The A188 genotype was used for these experiments rather than B73 due to the fact that A188 plants are largely successful when processed through tissue-culture whereas B73 performs poorly. In the analysis of these samples we found evidence for consistent alterations of DNA methylation in tissue-cultured individuals relative to controls (Stelpflug et al., 2014). The DMRs from both studies were combined to generate a non-redundant list of DMRs from tissue culture or abiotic stress. We further filtered this list to remove all regions that exhibit >0.25 difference between the B73 control average and the A188 average control to remove regions that might have genetic variation or genotype-specific differences in DNA methylation. The combined clustering of the DMRs from both abiotic stress and tissue-culture provide evidence for consistent changes within the tissue-culture samples. However, the A188 controls and B73 controls are interspersed with the individuals subjected to heat, cold or UV stress (Figure 5). This analysis suggests that we can detect treatment specific effects (tissue-culture) but that the abiotic stresses used in this study did not have substantial treatment effects.

## Discussion

Our experiment was designed to address two potential hypotheses about DNA methylation changes in response to abiotic stress. One hypothesis is that a particular stress will result in consistent DNA methylation alterations. The second hypothesis is that abiotic stress will result in increased variation in DNA methylation. In both cases the expectation is that these changes in DNA methylation will be mitotically heritable and maintained through the life of the plant. Prior to discussing our findings relative to these specific hypotheses it is worthwhile to consider the limitations of our study.

### Limitations to study design and interpretation

One limitation is that we are only studying mitotically heritable changes. The tissue that was used (last adult leaf) should be composed of mitotically derived descendants of cells that were still located within the meristem during the stress treatments. It is possible that treatments could result in DMRs that are only transiently formed or only “remembered” in meristematic cells and therefore would not be present in the tissue we sampled. This would reduce our power to detect methylation changes, as they would be present only in some of the cells.

A second limitation arises from the methodology used to profile DNA methylation. Compared to whole-genome bisulfite sequencing (WGBS) the meDIP-array profiling is relatively low-cost and still produces genome-wide scans. The lower cost enabled us to profile a greater number of individuals in this experiment. However, this method also has reduced resolution to detect DMRs, especially if DNA methylation changes are context-specific. There are many possible ways that DNA methylation could change in response to a treatment and we will consider our power to detect these changes in homozygous or heterozygous conditions.

A genomic region that begins as completely unmethylated and then gains DNA methylation in response to abiotic stress (or stochastic events) may be relatively easier to detect, especially in the heterozygous condition as there would be a strong gain of meDIP signal. Initially, these events are likely to be *de novo* DNA methylation events involving small RNA targeting of a DRM methyltransferase (Law and Jacobsen, 2010; Matzke and Mosher, 2014). This will result in moderate levels of DNA methylation in all sequence contexts. If there is substantial gain in DNA methylation then these regions might be detected as they would be effectively immunoprecipitated. However, if the *de novo* methylation level is low then these regions may not be strongly enriched following meDIP and may not provide enough signal for detection. *De novo* methylation of a region is often followed by stable maintenance of DNA methylation only in CG and CHG contexts catalyzed by MET1 and CMT enzymes, respectively (Law and Jacobsen, 2010). This maintenance DNA methylation often results in quite high levels of CG and CHG methylation and if some plants have triggered this pathway while others have not then this difference would likely be detectable in the experimental procedures we have used.

Losses of DNA methylation might be more difficult to detect, especially in the heterozygous condition. Genomic regions that begin with high levels of DNA methylation on both alleles could lose DNA methylation in one or both alleles. These losses could occur passively due to failure of maintenance DNA methylation activities or could occur actively through action of demethylase enzymes such as ROS1 (Zhang and Zhu, 2012). For both passive and active losses of DNA methylation it is quite possible for events to occur on one allele and not the other. This would result in heterozygosity for DNA methylation levels and these are difficult to detect using meDIP-array analysis. Since both fully methylated and heterozygote individuals contain methylated DNA at the locus you will recover meDIP signal in both and the quantitative differences in signal may not meet our criteria.

The gain, or loss, of CHH methylation may be difficult to detect using the meDIP approach. If two individuals both contain highly methylated DNA in the CG and CHG context at a particular locus and only differ in one having CHH methylation there are not likely to be differences in meDIP signal. The DNA from both individuals will be effectively immunoprecipitated and the extra CHH in one individual likely won't result in significantly more immunoprecipitation or signal. This means that our approach will struggle to identify most examples of changes in targeting of *de novo* methylation. Only in cases that go from completely unmethylated DNA to partially methylated CHH (as well as CG and CHG) will we potentially detect the DMR.

Despite the limitations described above, it is worth noting that our approach certainly would be able to detect major changes in DNA methylation patterns. If there was substantial perturbation of genomic DNA methylation this experiment would be able to detect those changes. In addition, if specific genomic regions went from being completely unmethylated to having moderate levels of DNA methylation this would have been readily detected. Similarly, if genomic regions had gone from moderate levels of DNA methylation to being unmethylated at both alleles this also would have been readily detected.

### Evaluating evidence for consistent stress-induced methylome perturbations

One hypothesis is that a particular stress, such as heat, would consistently result in specific changes to the methylome. There is evidence that this occurs in tissue-culture in both rice (Stroud et al., 2013) and maize (Stelpflug et al., 2014). However, in our analysis of cold, heat and UV-stressed maize seedlings we did not find evidence for consistent methylome perturbations. There are very few, if any, DMRs that are consistently observed in multiple individuals subjected to the same stress. Clustering of overall DNA methylation profiles do not find evidence for relationships of different individuals subjected to the same stress with the exception of plants subjected to tissue culture (Figure 5). This may not be that surprising. In order for consistent changes to occur in multiple individuals it would require that DNA methylation, or demethylation, machinery be targeted to specific genomic locations in response to particular environmental cues. Consistent changes to specific loci would also be expected to be homozygous as the methylation machinery would likely be targeted to both alleles. These types of changes should be easier to detect both due to the consistent presence in multiple individuals (providing greater statistical power) and due to the homozygous change (providing greater detection power). However, we did not observe clear evidence for consistent DNA methylation changes following the environmental conditions used in this study.

### Evaluating evidence for increase rate of changes in methylome following stress

A second hypothesis is that environmental stresses may result in increased stochastic variation for DNA methylation patterns. Stress would destabilize the methylation, or demethylation, machinery and this would essentially result in higher “epimutation” rates in stressed plants. This is a more difficult hypothesis to evaluate within the limitations of the current study. Stochastic epimutations are likely to be heterozygous and we would have limited power to detect these changes, especially for DNA methylation losses on only one allele. In addition, due to variance in the signal:noise ratio for the experimental data for different individuals the simple count of the number of DMRs per individual can be difficult to interpret. We did find more DMRs in the stressed plants than in the control plants. However, there are more stressed plants that were evaluated and the control plants were compared to the control average (which they contribute to). One interesting observation is that the relative frequency of hypermethylation events and hypomethylation events differs in the stress and control plants. The stressed plants have an excess of hypermethylated events. These events should be easier to detect in our experimental design and may indicate increased rates of DNA methylation gain in stress plants. Overall, our experiments suggest that there is not a major change in the rate of stochastic methylation changes but do suggest that there might be some differences. The recent study by Jiang et al. (2014) similarly found slight changes in both epimutation and mutation rate in Arabidopsis plants subjected to abiotic stress. Experiments that sample larger numbers of both control and stressed plants will be important in determining the exact rate for increased stochastic methylation changes.

### Conflict of interest statement

The authors declare that the research was conducted in the absence of any commercial or financial relationships that could be construed as a potential conflict of interest.
